# Impact of the COVID-19 pandemic on communal religious worshippers’ mental health and the benefits of positive religious coping

**DOI:** 10.1016/j.heliyon.2024.e39093

**Published:** 2024-10-09

**Authors:** Rebecca F. Baggaley, Kai Man Alexander Ho, John Maltby, Timothy C. Stone, Áine Hoga, Christopher Johnson, Robert Merrifield, Laurence B. Lovat

**Affiliations:** a Department of Population Health Sciences, University of Leicester, University Road, Leicester, LE1 7RH, United Kingdom; b Department of Targeted Intervention, University College London, Charles Bell House, 43-45 Foley Street, London, W1W 7TY, United Kingdom; c Wellcome/EPSRC Centre for Interventional and Surgical Sciences (WEISS), University College London, Charles Bell House, 43-45 Foley Street, London, W1W 7TY, United Kingdom; d Centre for Psychological Health and Development, Psychology and Vision Sciences, College of Life Sciences, University of Leicester, Leicester, United Kingdom; e Dynaimx, 71-75 Shelton Street, London, WC2H 9JQ, United Kingdom

**Keywords:** COVID-19, Religion, Depression, Anxiety, Wellbeing, Lockdown, Religiosity, Factor analysis

## Abstract

**Background:**

In the United Kingdom, onsite religious services were halted during COVID-19 lockdowns, which were followed by various levels of restrictions on communal worship including social distancing, mandatory wearing of face masks, adequate ventilation and a ban on congregational singing and chanting. The aim of our study was to evaluate the impact of closures and changes within places of worship in response to the first lockdown in 2020, to assess the effect of the pandemic on religious practice and worshippers’ wellbeing and religious coping.

**Methods:**

Participants were regular worshippers in the UK, recruited through an online survey using convenience sampling. Respondents were asked about their attitudes to changes to places of worship in the UK and their wellbeing and mental health, including assessment of their risk of depression and anxiety using the Patient Health Questionnaire (PHQ-9) and Generalised Anxiety Disorder Assessment (GAD-7) measures. Questionnaires were completed August to November 2020.

**Findings:**

939 participants were included in the analysis. Median age was 52.7 years and 66.1 % were female. 80.7 % identified as Christian. 165 (19.3 %) had mild, 45 (5.3 %) moderate, 25 (2.9 %) moderately severe and 10 (1.2 %) severe depression, and 192 (22.5 %) had mild, 55 (6.4 %) moderate and 27 (3.2 %) severe anxiety, according to PHQ-9 and GAD-7 scores, respectively. Nearly half (46.4 %) reported that their mood and anxiety levels had worsened and 16.6 % reported that they felt the things they were doing in their lives were not worthwhile. The vast majority of respondents (92.7 %) reported that prayer had helped them cope with the way they had felt during lockdown: 29.2 % and 47.0 % reported that it helped moderately and a great deal, respectively. This 76.2 % had significantly lower levels of moderate/severe depression and anxiety (adjusted odds ratio: depression 0.37 (95%CI 0.22–0.63), anxiety 0.52 (95%CI 0.31–0.88).

**Interpretation:**

Our study demonstrates the significant impact of COVID-19 on communal worshippers’ mental health and reinforces the benefits of positive religious coping during the first UK lockdown. Barriers to communal worship participation during lockdowns, including access to appropriate technology, need to be recognised and facilitators identified.

## Highlights

• COVID-19 has had a significant impact on communal worshippers' mental health.
• Prayer helped respondents cope with the way they felt during the first UK lockdown.
• This reinforces the benefits of positive religious coping during time of crisis.

## 1 Introduction

The COVID-19 pandemic altered the landscape of religious worship in the United Kingdom (UK). Measures enacted to curb the pandemic's spread, including nationwide lockdowns, the introduction of the tiered system, and various restrictions on religious gatherings, led to drastic changes in traditional worship practices. Onsite religious services were halted in March 2020 due to the rising incidence of SARS-CoV-2 infections. They resumed in July 2020 but with a number of restrictions on traditional worship activities, including prohibition of congregational singing and chanting, reduced congregation sizes, social distancing and obligatory mask-wearing [1]. The suspension of onsite religious services and the subsequent reopening with limitations substantially transformed worship routines.

The COVID-19 pandemic also amplified mental health challenges including depression, anxiety, stress, panic attacks, and sleep disorders, although studies have suggested that this effect was transient and prevalence of these conditions returned to pre-pandemic levels amongst most population subgroups [2,3]. It affected virtually all spheres of life, including physical health, the economy, education, work, mobility, isolation from loved ones, scarcity of consumer goods, paralysis of health care systems, economic uncertainty, including redundancies and bankruptcy, and remote work/education without physical contact with other people [4]. All these factors, and many others, contributed to reduced quality of life and poorer mental health [5,6].

During challenging times, religious practices often intensify [7,8], providing solace, hope, and community for many, and the COVID-19 pandemic witnessed a change in religious engagement. In the first months of the pandemic, Google search data across 107 countries showed that “prayer” relative to all Google searches rose by 30 %, reaching the highest level ever recorded [8]. According to a 2020 survey, a quarter of British adults prayed for an end to the pandemic [9]. The rise in online services and innovative worship practices like neighbourhood prayer meetings in communities with limited internet access, such as the Haredi Jews in London meeting across back gardens and streets rather than in a synagogue [9], underscore the adaptability of religious communities in the face of adversity.

Religion and spirituality have been shown to improve mental health outcomes for individuals affected by a wide range of negative circumstances including serious illness, natural disasters and war [10]. In addition, places of worship play an important role in society, not only by providing spiritual guidance and expression, but also by bringing communities together.

Individuals may use a variety of coping strategies during stressful situations, including religious coping. Religious coping can involve a variety of religious practices and may help an individual to reinterpret a stressor (for example, “it is God's will”) or provide emotional support (from God and/or from religious communities) [4]. Religious coping may therefore improve mental health for individuals active in faith during the pandemic by providing comfort and hope, enabling them to perceive adversity positively and regulate fear [11].

**Fig. 1:**
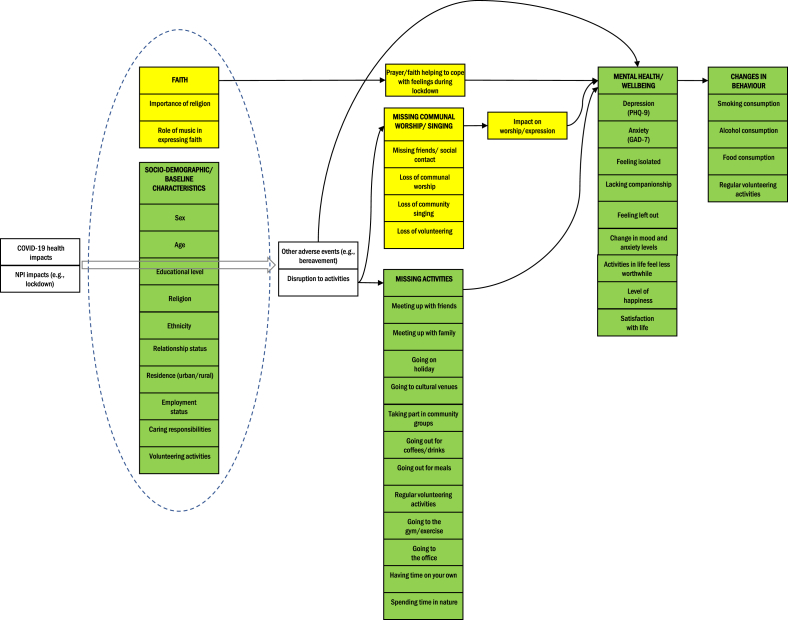
Theoretical framework illustrating the relationships between COVID-19 effects, missing communal worship activities and activities more generally, respondents' mental health and wellbeing, and consequent coping mechanisms and changes to behaviour. NPI – non-pharmaceutical intervention.

The CONFESS study, an online questionnaire-based study, aimed to understand these changes in religious worship and how they affected the mental health and wellbeing of individuals active in faith during the first UK COVID-19 lockdown. Besides assessing the comfort and acceptability of mask-wearing during worship activities [12], this study also aimed to gain insights into the wider impacts of COVID-19 on religious practice and the subsequent effects on individuals' lives and mental wellbeing. By using a range of survey measures and recruitment strategies, the study aimed to draw participants from diverse religious backgrounds and assess various aspects of their religious activities and mental health status during the pandemic. The aim of the current analysis is to shed light on the changes in religious practices due to the COVID-19 pandemic and how faith-based coping mechanisms, as outlined in our theoretical framework (Fig. 1), might have influenced mental health outcomes. Such insights may be crucial for developing future strategies to support religious communities during crises.

## 2 Methods

### 2.1 Study design

CONFESS (COvid aNd FacE maSkS [13]) is a cross-sectional study designed to assess the impact of the COVID-19 pandemic on various aspects of religious practice using an online questionnaire. Participants were required to be at least 18 years old and able to understand English. Participation was voluntary and respondents were recruited via a convenience sampling technique using word of mouth, targeted advertising through religious institutions, social media such as Facebook and WhatsApp, and publicity with the British Broadcasting Corporation (BBC), which featured the study in the national news bulletin and religious affairs programme [14]. Particular efforts were made to recruit participants from a range of religious backgrounds including Christian, Muslim, Jewish, Hindu and Buddhist by contacting specific groups, such as through Facebook (see Table S1, Supplementary Information File 1 for a list of groups contacted). In addition, religious leaders were approached directly from churches, mosques, synagogues and Hindu, Buddhist and Hare Krishna temples. Respondents accessed the consent form and questionnaire through a weblink. Approximately 75 % of respondents accessed this link through an online BBC article promoting the study [14]. Recruitment and questionnaire completion occurred between August and November 2020, with the last questionnaire completed on November 5, 2020.

### 2.2 Declarations

This study was approved by the Research Ethics Committee of University College London September 2020, with ethics approval reference 14223/002. Participants completed informed consent for the online questionnaire.

### 2.3 Survey measures

The CONFESS study comprised an online, self-administered questionnaire including items concerning demographic characteristics, including age, sex, religion and ethnic background; and questions relating to COVID-19 and its control measures, including changes in worshipping practice due to COVID-19; acceptability and comfort of face mask wearing during communal worship and singing during worship; awareness and understanding of government guidelines regarding COVID-19 and compliance of participants’ place of worship with these guidelines.

In addition, respondents were asked about how COVID-19 restrictions on communal worship had affected them in terms of income, mood, socialising, and eating habits. The survey included 12 items on the extent to which individuals “missed” certain activities during the first lockdown restrictions, such as meeting up with friends and family and going out for meals. The survey also included eight items relating to faith (e.g., whether respondents considered themselves active in organised religion).

Standardised measures of depression and anxiety (the 9-item Patient Health Questionnaire (PHQ-9) [15] and the 7-item Generalised Anxiety Disorder Assessment (GAD-7) [16,17], respectively) were included to quantitatively assess mental health outcomes. PHQ-9 consists of the nine criteria upon which the diagnosis of DSM-IV depressive disorders is based and is a widely used depression screening instrument in nonpsychiatric settings. Respondents are asked to rate each of the items on a scale of 0–3 on the basis of how much a symptom has bothered them over the last 2 weeks (0 = not at all, 1 = several days, 2 = more than half the days, 3 = nearly every day). The summed score for all nine items gives a total score ranging from 0 to 27. PHQ-9 scores of 0–4, 5–9, 10–14, 15–19, and 20–27 have been validated to represent no depression, mild, moderate, moderately severe, and severe depression, respectively [15].

GAD-7 uses the same rating scores as PHQ-9 (0–3). The summed score for all seven items gives a total score ranging from 0 to 21. GAD-7 scores of 5–9, 10–14, and 15–21 are taken as the cut-off points for mild, moderate and severe anxiety, respectively. A complete list of PHQ-9 and GAD-7 items is shown in Table S2, Supplementary Information File 1.

The 12 questionnaire items representing missing activities were derived from the dataset used by the UCL COVID-19 Social Study [18]. The eight items representing faith were newly-devised item sets. There are numerous studies that identify the spiritual needs of individuals, most of which are validated in patient groups [19]. However, none of these identified methods or forms of prayer and therefore we designed our own questions to ensure we obtained the appropriate information for our study.

To supplement quantitative data, the study also included open-ended questions that provided deeper insights into how religious individuals navigated and adapted to these changes. It was also possible to skip questions so not all participants answered every question.

The CONFESS study was designed in the first few months of the COVID-19 pandemic as it affected the UK, implementing the questionnaire by August 2020. Its primary aim was to explore the impact and acceptability of face mask wearing and other non-pharmaceutical interventions, such as social distancing, for communal worshippers as services and ceremonies were restarted in summer 2020. The study provided an important opportunity to learn more about the impact of lockdown on communal worshippers' lives more generally, including their health and wellbeing. We did not develop an *a priori* theoretical framework of the impact of pandemic-related disruption to communal religious worshippers' mental health because our research was more exploratory in nature. However, through preliminary analysis of questionnaire item outcomes and the insights drawn from open-ended questions, we developed a theoretical framework illustrating the relationships between COVID-19 effects (both health effects and disruptions due to non-pharmaceutical interventions, including lockdowns), missing communal worship activities and activities more generally, respondents’ mental health and wellbeing, and consequent changes to their behaviour. This framework shaped the structure of our analysis and is illustrated in Fig. 1.

### 2.4 Data analysis

Statistical processing of questionnaire data: Data were transferred to an MS Excel spreadsheet before quantitative data were analysed using R software version 4.0.4. We followed STROBE guidelines in the reporting of this manuscript [20].

Statistical methods: We performed a primarily descriptive analysis, plus univariate and multivariable (adjusted) logistic regression to identify characteristics which may predict high risk of depression and anxiety, expressed as unadjusted and adjusted odds ratios (aORs) with 95 % confidence intervals (95%CI) (adjusting for the following sociodemographic variables: sex, age, highest educational level, religion, ethnicity, relationship status, place of residence and employment status). A p-value of ≤0.05 was taken as statistically significant.

For the 12 items relating to missing activities and the eight items relating to faith, we had no *a priori* hypotheses about factors or patterns between these items. We therefore carried out exploratory factor analysis on each item set to understand the relationships between the items within each set and the underlying factors that the items may have in common. The number of factors for each item set was determined by Parallel Analysis, using principal-axis factoring due to the non-parametric nature of the data and oblimin rotation, to determine the latent structure using half the data, determined by splitting the data in half using random numbers. We performed Confirmatory Factor Analysis using the second half of the data to provide structural validity of the scale. Goodness of fit of the models was measured by Goodness of Fit (GFI), Adjusted Goodness of Fit (AGFI) and Normed Fit (NFI) indices using unweighted least squares, as appropriate for non-parametric data. For each identified factor, its correlation with depression (PHQ-9) and anxiety (GAD-7) scores was evaluated using Pearson's Correlation Coefficient.

Qualitative analysis: We performed thematic analysis of open-ended questions to complement the quantitative analysis.

### 2.5 Role of funding source

The funders of the study had no role in study design, data collection, data analysis, data interpretation, or writing of the report.

## 3 Results

We surveyed a total of 1063 individuals, of whom 939 (88.3 %) completed the questionnaire and were included in the final analysis. A portion of the respondents (59.1 %) also provided responses to open-ended questions, which asked for feedback on the questionnaire and the study in general.

### 3.1 Demographics

Demographic characteristics of included respondents are shown in Table 1. Median age was 52.7 years and two-thirds (66.1 %) were female. 845 (90.0 %) completed at least undergraduate-level education. The majority of respondents were Christian (n = 758, 80.7 %), followed by Jewish (n = 145, 15.4 %) and were of white ethnicity (n = 869, 92.5 %). 789 (84.0 %) of participants lived in urban areas. Of the 831 (88.5 %) respondents who gave valid postcode data, 305 (36.7 %) lived in London, 122 (14.7 %) lived in the East of England and 105 (12.6 %) lived in Southeast England (Fig. S1, Supplementary Information File 1 shows the distribution of respondents across the UK). Most participants (779/939, 83.0 %) reported neither having had suspected nor confirmed COVID-19 infection. Further characteristics of respondents can be found in Ho et al. [12].

**Table 1:** Demographic characteristics of survey participants (n = 939) and their association with depression (PHQ-9 score) and anxiety (GAD-7 score).

Characteristic			Moderate/severe depression (PHQ-9^2^)			Moderate/severe anxiety (GAD-7^3,4^)		
		Participants (%)	x/n (%)	aOR (95%CI)	p value	x/n (%)	aOR (95%CI)	p value
**Demographic characteristics**								
**Sex**^a^	Female	620 (66.1 %)	54/573 (9.4 %)	–	–	59/573 (8.2 %)	–	–
	Male	318 (33.9 %)	26/280 (9.3 %)	1.07 (0.62–1.91)	NS	23/280 (10.3 %)	1.52 (0.87–2.74)	NS
**Age**^b^	Median age (range), years^c^	52.7 (18–85)	–	-c	-c	–	**-**c	**-**c
	18–34 years	161 (17.7 %)	21/147 (14.3 %)	–	–	30/147 (20.4 %)	–	–
	35–64 years	585 (64.1 %)	52/535 (9.7 %)	0.89 (0.43–1.91)	NS	40/535 (7.5 %)	**0.35 (0.17–0.70)**	**0.003**
	≥65 years	166 (18.2 %)	6/148 (4.1 %)	**0.27 (0.06–1.03)**	**0.062**	10/148 (6.8 %)	**0.21 (0.06–0.71)**	**0.013**
**Highest educational**	Undergraduate degree or professional qualification	470 (50.1 %)	37/434 (8.5 %)	–	–	36/434 (8.3 %)	–	–
	Postgraduate degree	375 (39.9 %)	34/338 (10.1 %)	1.17 (0.67–2.04)	NS	38/338 (11.2 %)	1.71 (0.99–2.99)	0.057
**level**	A-levels or equivalent/post-16 vocational course	72 (7.7 %)	7/63 (11.1 %)	1.23 (0.46–2.94)	NS	6/63 (9.5 %)	1.17 (0.40–2.97)	NS
	GCSE/CSE/O-levels or equivalent/no qualifications	22 (2.3 %)	2/18 (11.1 %)	1.25 (0.18–5.21)	NS	2/18 (11.1 %)	1.61 (0.23–6.71)	NS
**Religion**	Christian	758 (80.7 %)	70/705 (9.9 %)	–	–	67/705 (9.5 %)	–	–
	Jewish	145 (15.4 %)	8/127 (6.3 %)	0.71 (0.29–1.56)	NS	13/127 (10.2 %)	1.48 (0.70–2.97)	NS
	Other^d^	36 (2.8 %)	2/21 (9.5 %)	0.87 (0.12–3.80)	NS	2/21 (9.5 %)	0.79 (0.11–3.48)	NS
**Ethnicity**	White Britis	792 (84.4 %)	68/724 (9.4 %)	–	–	68/724 (9.4 %)	–	–
	Other White background	77 (8.2 %)	5/71 (7.0 %)	0.72 (0.24–1.82)	NS	8/71 (11.3 %)	1.09 (0.43–2.48)	NS
	Asian/Asian British	26 (2.8 %)	2/21 (9.5 %)	0.73 (0.10–3.34)	NS	3/21 (14.3 %)	0.98 (0.18–4.01)	NS
	Black/African/Caribbean	12 (1.3 %)	2/9 (22.2 %)	2.13 (0.28–10.92)	NS	0/9 (0.0 %)	–	–
	Mixed/Multiple ethnic groups	16 (1.7 %)	2/15 (13.3 %)	1.79 (0.25–7.88)	NS	2/15 (13.3 %)	1.04 (0.15–4.66)	NS
	Other	15 (1.6 %)	1/13 (7.7 %)	1.05 (0.05–6.42)	NS	1/13 (7.7 %)	1.00 (0.05–5.97)	NS
**Relationship**	In a relationship/married and cohabiting	686 (73.1 %)	45/628 (7.2 %)	–	–	48/628 (7.6 %)	–	–
**status**	In a relationship/married but living apart	36 (3.8 %)	5/32 (15.6 %)	2.16 (0.66–6.00)	NS	5/32 (15.6 %)	1.47 (0.44–4.16)	NS
	Single, divorced or widowed	60 (6.4 %)	6/54 (11.1 %)	1.59 (0.54–4.05)	NS	4/54 (7.4 %)	1.08 (0.30–3.06)	NS
	Single, never married	157 (16.7 %)	24/139 (17.3 %)	**2.37 (1.18–4.72)**	**0.015**	25/139 (18.0 %)	1.71 (0.85–3.40)	**NS**
**Place of residence**	City/town	789 (84.0 %)	68/722 (9.4 %)	–	–	68/722 (9.4 %)	–	–
	Village/rural dwelling	150 (16.0 %)	12/131 (9.2 %)	1.16 (0.54–2.32)	NS	14/131 (10.7 %)	1.49 (0.71–2.99)	NS
**Employment status**	Employed (full-time)	374 (39.8 %)	42/340 (12.4 %)	–	–	42/340 (12.4 %)	–	–
	Employed (part-time)	175 (18.6 %)	10/165 (6.1 %)	0.51 (0.22–1.11)	NS	8/165 (4.8 %)	**0.36 (0.13–0.84)**	**0.026**
	Self-employed	113 (12.0 %)	9/98 (9.2 %)	0.83 (0.34–1.85)	NS	7/98 (7.1 %)	0.78 (0.29–1.87)	NS
	Retired	193 (20.6 %)	10/173 (5.8 %)	0.64 (0.22–1.66)	NS	14/173 (8.1 %)	0.91 (0.33–2.38)	NS
	Student (university/school)	35 (3.7 %)	4/30 (13.3 %)	0.68 (0.17–2.22)	NS	5/30 (16.7 %)	0.73 (0.21–2.22)	NS
	Other^e^	49 (5.2 %)	5/47 (10.6 %)	0.85 (0.27–2.27)	NS	6/47 (12.8 %)	1.09 (0.36–2.89)	NS
**Other characteristics**								
**Caring**	None	482 (51.3 %)	39/396 (9.8 %)	–	–	45/396 (11.4 %)	–	–
	Children	278 (29.6 %)	21/278 (7.6 %)	0.78 (0.40–1.44)	NS	21/278 (7.6 %)	0.45 (0.20–0.92)	0.040
	Grandchildren	66 (7.0 %)	8/66 (12.1 %)	1.52 (0.65–3.20)	NS	6/66 (9.1 %)	1.97 (0.89–4.07)	0.079
	Elderly relatives/friends	183 (19.5 %)	15/183 (8.2 %)	0.82 (0.44–1.52)	NS	9/183 (4.9 %)	0.95 (0.50–1.78)	NS
	People with long-term conditions/disabilities	87 (9.3 %)	9/87 (10.3 %)	2.57 (0.99–6.13)	0.040	12/87 (13.8 %)	1.46 (0.50–3.69)	NS
**Volunteering**	About the same	369 (45.1 %)	29/369 (7.9 %)	–	–	29/369 (7.9 %)	–	–
**since start of**	Less than usual	337 (41.1 %)	38/337 (11.3 %)	1.54 (0.90–2.66)	NS	41/337 (12.2 %)	**1.74 (1.01–3.01)**	0.046
**pandemic**	More than usual	113 (13.8 %)	11/113 (9.7 %)	1.28 (0.56–2.77)	NS	11/113 (9.7 %)	1.46 (0.63–3.17)	NS
**Adverse event**	No	664 (70.7 %)	49/578 (8.5 %)	–	–	50/578 (8.7 %)	–	–
**since start of pandemic**^f^	Yes	275 (29.3 %)	31/275 (11.3 %)	1.29 (0.77–2.15)	NS	32/275 (11.6 %)	1.27 (0.75–2.13)	NS

aOR – adjusted odds ratio; NS – not significant (p > 0.10); OR – odds ratio; uOR – unadjusted odds ratio; 95%CI – 95 % confidence interval.

a The variable sex was defined as sex at birth, which agreed in all cases with current gender identity, where the latter question was completed. One respondent reported that their gender-identity was different from their birth-assigned gender, but sex at birth and current gender identity were the same for this individual. Missing data: n = 1 (0.1 %).

b Missing data: 27/939 (2.9 %).

c Age included in the multivariable logistic regression model as a categorical variable only.

d “Other” category includes Muslim, Buddhist, Hindu, Sikh, any other religion and no religion (numbers of respondents too small to report separately).

e “Other” category includes homemaker, full-time parent, carer, job seeker and those unable to work due to disability (numbers of respondents too small to report separately).

f Referent category is respondents not reporting caring responsibilities for that category of individuals (rather than the “None” i.e., no caring responsibilities category).

### 3.2 Faith: importance of religion, community and music-making

**Fig. 2:**
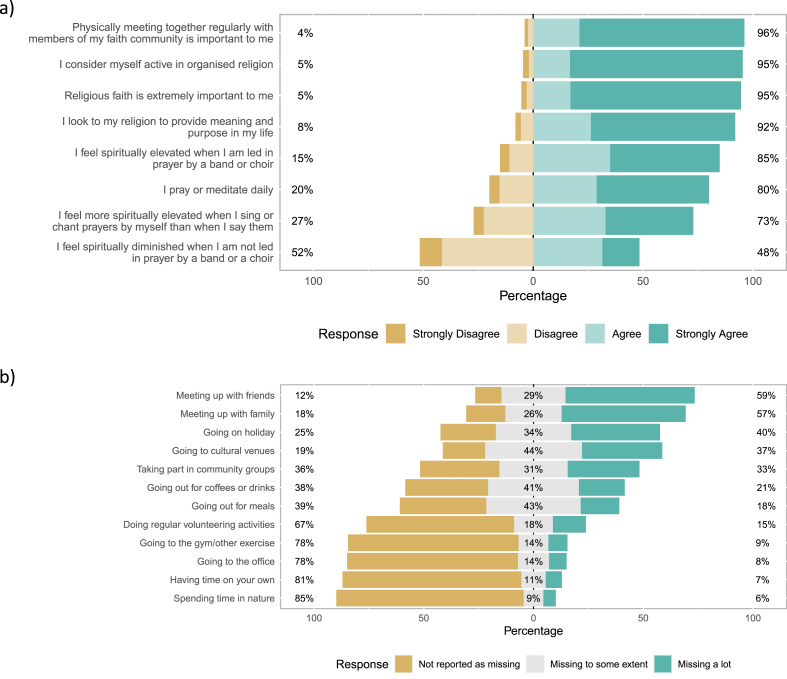
Responses to questionnaire items relating to a) faith, in terms of importance of religion and the role of music in expressing faith for study participants, and b) missing activities i.e., whether study participants were missing various aspects of life a lot or to some extent during the first UK COVID-19 lockdown.

Questions relating to faith are shown in Fig. 2a. The vast majority (95 %) of respondents considered themselves active in organised religion. Only 3 % did not consider themselves to be religious. Emerging themes regarding the impact of COVID-19 on communal worship taken from analysis of responses to open-ended questions are shown in Table 2, accompanied by illustrative quotes. While spirituality and faith were considered extremely important (78 % strongly agreed that religious faith was important to them, Fig. 2a), respondents also expressed the importance of being with others (Table 2 quotes 1–5; 96 % felt that physically meeting with members of their faith community was important). Responses to open-ended questions suggested that it was important for respondents both to worship and pray together (Table 2 quotes 9, 14), but most responses on this theme discussed the important social aspects of communal worship (quotes 12–17). The value of music during communal worship was also highlighted: 85 % felt spiritually elevated when led in prayer by a band or choir, and many respondents expressed the importance of joining in with music making: a way to share worship and express faith (Table 2 quotes 3–6, 8, 10, 11).

**Table 2:** Quotations of respondents, illustrating the emerging themes.

A) COVID-19 impact on communal worship and singing	
**Loss of communal worship**	
1	*“Communal singing and worship are central to my faith and I feel the loss of it greatly. For me, it's probably the hardest thing about current restrictions.”*
2	*“Congregation worship is a very important part of my life.”*
**Loss of singing during communal worship – impact on worship/expression**	
3	*“I miss singing hymns and other Christian songs in church enormously – they're a part of my self-identity and a way of sharing in worship with others.”*
4	*“Singing is a key part of worship and the experience of singing with others releasing peace and joy.”*
5	*“Singing was a massive part of my expression of faith, and of my day-to-day life.”*
**Loss of singing during communal worship – impact on wellbeing**	
6	*“Not being able to sing has had a direct impact on my mental health.”*
7	*“The lack of singing at church is a massive negative impact on my life.”*
8	*“I have felt real grief over the loss of singing. Singing was integral to my ability to worship, and provided me with joy, purpose, companionship, volunteering opportunities, and physical and mental well-being both within and beyond religious contexts. I felt guilt because I was grieving for the loss of "mere" singing when so many people were grieving for sickness and death of loved ones, or loss of livelihood. I felt guilt that my faith and my prayer life, both of which had been very much bound up in communal worship, prayer or study, seemed to vanish like smoke when churches were closed.”*
**Community singing – joining in with others**	
9	*“Communal worship is very important to me. I really miss hearing a church singing in unity together. Whilst church continues and we are able to reach most people either online or within the building there is a real sense that we are missing an important part when we can't worship together.”*
10	*“Sung worship with others in church is different to sung worship alone at home and I lament not being able to do this due to Covid.”*
11	*“As for singing, there is no set expectation in my Christian faith for a certain amount or type of singing, but if you can imagine a football match with no songs or Christmas without songs, you can begin to understand how we are feeling. We can worship in other ways and our faith does not suffer but our hearts are sad because we love to sing together.”*
**Loss of social contact**	
12	*“I am part of the team that puts on refreshments at synagogue and I do miss that; they are great social occasions which are just as important as praying in my opinion.”*
13	*“At my church the loss of the worshipping community; no socialising after church with tea and coffee, no community meals, social events. People don't even stay around outside after to chat in groups. Something fundamental is missing. The personal contact is diminishing which is quite hard.”*
14	*“I used to love going to church, I loved to sing and to pray together, to spend time with people over a meal at church (after the service) and to get to know new people. It helped me connect with God, to grow in my faith and to build relationships which were good for me and also helped me cope with the loneliness of being single in a big city. Now church feels like a chore … I find myself preferring to watch it online but this isn't the same – I feel less connected to God, find it harder to engage with the teaching, I miss communal prayer and worship and I miss other people. I still love Jesus but I am really struggling with church and feel quite sad about it every time I go!”*
15	*“Not being able to socialise after Mass is a major downside of the pandemic. I miss the interaction with my church friends and my community activity friends.”*
16	*“Prayer (private and corporate) is part of my faith, but worship and social interaction is a greater part and this has been significantly affected by the Covid restrictions.”*
17	*“Our congregation is older and many live alone. This has been isolating.”*
**Loss of volunteering opportunities**	
18	*“I badly miss volunteering with young children and playing music with my friends.”*
**Benefits of online worship and singing**	
19	*“Although my place of worship has reopened I have chosen not to go because I am not able to sing … At present I feel I can worship better listening to the service at home where I can join in with pre-recorded hymns and sing in worship to God. It makes me feel more connected and joyful.”*
20	*“Virtual prayer and virtual faith/service type meetings using technology platforms … have been tremendously sustaining, in fact the emergence of contact and connection through virtual platforms has really offered surprising opportunity and deepening of community.”*
21	*“For me, not being able to sing in church has taken the 'joy' out of worship. Worship feels 'distanced' and 'flat'. I preferred Zoom Worship to how we currently worship in church, as we could sing and felt we were all participating together and there was always 'chat' afterwards.”*
22	*“My church very quickly offered online worship and still is and it is amazing and such a joy our priest is quite young and really knows how to use technology to great effect. We are actually a bigger faith community now because lots of folk are logging on from other churches because their churches aren't offering this. it's been a bit of a revelation actually and the lord is present in all media.”*
**Barriers to online worship – practical issues**	
23	*“Online worship can be difficult for someone who doesn't have access to modern technology/strong wifi.”*
24	*“Because I am orthodox Jewish I cannot join in much of the online worship organised by synagogues since I don't use electric communication devices on sabbath or festivals and my informal Jewish prayer group has decided not to meet face to face. So I have been enjoying God's creation in the local park instead.”*
25	*“Zoom church services are too frustrating as it keeps freezing during songs*.*”*
**Online worship is not a substitute for communal worship**	
26	*“Doing services at home is okay but lacks the sense of community. My children are also missing out. They find it hard to connect with online services.”*
27	*“We have online church but for many it's hard to engage with as its just another screen in some ways.”*
28	*“[Weekly online services] have helped in maintaining corporate worship but do not come close to the atmosphere we had before lockdown, and it is much harder for children and older people to engage.”*
29	*“We have Prayer meetings over Zoom however it is not the same or as intuitive as in person. I miss the hugs with friends at church and body language when you are praying for someone and see the release prayer brings from burdens and when Holy Spirit is working.”*
**Beneficial side effects of preventing communal worship**	
30	*“I have spent more time on religious learning rather than prayer – this provides spiritual benefits too!”*
31	*“So much community engagement has ceased in the pandemic, especially with young families … Paradoxically, less time in church evening meetings, planning and so on has allowed more time for personal prayer and study, which has been lovely. But really my faith thrives in relationships and that continues to be a great gap in weekly life.”*
**B) Returning to communal worship**	

***Benefits of returning to communal worship***	
32	*“I am so joyful that services although much shorter have started again. The fellowship of being with other believers and part of God's community here on earth is unlike any other in my life.”*
33	*“I found that the lack of singing during lockdown made me feel less connected to God during the service. Yesterday, it felt like things were starting to get back to normal and I could feel God with us a lot more.”*
***Feelings of safety/reassurance***	
34	*“Of all the places I have been to since the start of Covid-19 (public transport, shops, cafés, travelling by place during the summer, and others) – my Church has by far treated social distancing and disinfecting most seriously. In fact, I feel that going to Church has made me more careful because our Priest encourages us to abide by the government's recommendations. I have treated this more seriously than say reminders on the tube and now I use hand gel more often – as a result of getting into that habit at Church.”*
35	*“I find myself unable to attend my own place of worship because I judge that the necessary precautions to guard against the spread of the virus are not being taken. I therefore attend another local church that is in my view "safe". This makes me feel quite depressed and angry at times.”*
**C) Singing as an expression of freedom**	

42	*“I hate the idea of anyone singing into a mask. Singing is about freedom.”*
43	*“There is usually a freedom in singing which would be affected by these restrictions.”*

### 3.3 Impact of COVID-19: missing activities and faith

Questions relating to missing activities during the first UK COVID-19 lockdown are shown in Fig. 2b. Respondents reported missing a range of activities, the most frequently reported being missing family (82.2 %) and friends (88.0 %). Relatively few respondents reported missing being on their own (18.5 %) or being at the office (22.2 %). Patterns of missing activities varied little by sex (activities missed stratified by sex shown in Fig. S2).

Factor Analysis of the missing activities item set suggested three factors of items that are interpretable (Table S3, Supplementary Information File 1). The first factor contains two items relating to missing friends and family (orange: relationships). The second factor contains five items relating to everyday activities such as being with nature and going to the gym (green: interests). The third factor contains two items that refer to missing culture and community (blue: identity).

Factor Analysis results suggested two factors for faith item sets, which align with the well-established distinction between religiosity and spirituality (Table S3). The first factor contains four items relating to faith importance, engagement and purpose, aligned with coming together religiously and praying daily (orange: religiosity). The second factor contains three items relating to the importance of engaging in spiritual activities such as choir membership, singing and prayer (blue: spirituality).

In open-ended questions, respondents expressed the impact of lockdown on their faith. They felt the loss of communal worship as a personal loss (Table 2 quote 1). Lack of singing during communal worship was expressed as having a negative impact on life (quote 7), affecting mental health and wellbeing (quotes 6, 8). One individual wrote that while their faith did not suffer, there was still sadness from not singing together (quote 11). Loss of personal and social contact through places of worship was frequently reported (quotes 12–17).

Many respondents discussed their experience with worship activities available online since the start of the pandemic. Many preferred attending online services, either because they felt free to sing (quotes 19, 21) or it facilitated social interaction (quote 20), but barriers to online participation were cited, including access to appropriate technology (quote 23) and intermittent broadband connection (quote 25). Furthermore, many expressed that online services were no substitute because they lacked the social interaction or atmosphere of physically being with others, with particular mention of the difficulty children and the elderly have with engaging with online services (quotes 26–29).

A minority of respondents reported beneficial side effects of places of worship closures: some of those involved in organising activities reported that time freed from planning and meeting allowed more time for personal prayer and religious learning (quotes 30, 31).

Table 3 summarises behaviour changes that respondents reported since the start of the pandemic. Respondents reported in approximately equal frequencies increases and reductions in smoking. Of those who drank alcohol, more (18.0 % of respondents) reported an increase in alcohol consumption since the start of the pandemic than those reporting a decrease (10.0 %). The same pattern was observed with food, with 24.1 % reporting consuming more food since the start of the pandemic and only 9.2 % reporting consuming less.

**Table 3:** Changes in behavioural characteristics and wellbeing of survey participants (n = 939) since the start of the COVID-19 pandemic and their association with PHQ-9 and GAD-7 scores.

			Moderate/severe depression (PHQ-9^2^)			Moderate/severe anxiety (GAD-7^3,4^)		
			x/n (%)	aOR (95%CI)	p value	x/n (%)	aOR (95%CI)	p value
Smoking consumption since the start of the pandemic	Non-smoker	861 (98.5 %)	77/832 (9.3 %)	–	–	80/832 (9.6 %)	–	–
	About the same	8 (0.9 %)	0/7 (0.0 %)	NA	NA	0/7 (0.0 %)	NA	NA
	Less than usual	3 (0.3 %)	0/3 (0.0 %)	NA	NA	1/3 (33.3 %)	4.89 (0.21–59.2)	NS
	More than usual	2 (0.3 %)	1/2 (50.0 %)	8.69 (0.28–26.1)	NS	0/2 (0.0 %)	NA	NA
Alcohol consumption since the start of the pandemic	Non-alcohol drinker	255 (29.2 %)	22/244 (9.0 %)	–	–	21/244 (8.6 %)	–	–
	About the same	374 (42.8 %)	27/361 (7.5 %)	0.90 (0.49–1.70)	NS	33/361 (9.1 %)	1.25 (0.68–2.34)	NS
	Less than usual	87 (10.0 %)	8/87 (9.2 %)	1.08 (0.42–2.56)	NS	8/87 (9.2 %)	1.15 (0.45–2.74)	NS
	More than usual	157 (18.0 %)	21/151 (13.9 %)	1.73 (0.86–3.47)	NS	19/151 (12.6 %)	1.68 (0.82–3.46)	NS
Food consumption since the start of the pandemic	About the same	583 (66.7 %)	29/566 (5.1 %)	–	–	41/566 (7.2 %)	–	–
	Less than usual	80 (9.2 %)	11/78 (14.1 %)	**3.12 (1.39–6.63)**	**0.004**	10/78 (12.8 %)	1.63 (0.72–3.39)	NS
	More than usual	211 (24.1 %)	38/200 (19.0 %)	**4.02 (2.36–6.91)**	**<0.001**	30/200 (15.0 %)	**2.16 (1.27–3.64)**	**0.004**
Volunteering since the start of the pandemic	About the same	369 (45.1 %)	29/369 (7.9 %)	–	–	29/369 (7.9 %)	–	–
	Less than usual	337 (41.1 %)	38/337 (11.3 %)	1.55 (0.92–2.65)	NS	41/337 (12.2 %)	**1.80 (1.07–3.06)**	**0.027**
	More than usual	113 (13.8 %)	11/113 (9.7 %)	1.26 (0.56–2.69)	NS	11/113 (9.7 %)	1.43 (0.63–3.04)	NS
Adverse event since the start of the pandemic^a^	No	664 (70.7 %)	49/578 (8.5 %)	–	–	50/578 (8.7 %)	–	–
	Yes	275 (29.3 %)	31/275 (11.3 %)	1.36 (0.82–2.23)	NS	32/275 (11.6 %)	1.30 (0.79–2.13)	NS
Since the lockdown for COVID started, have your mood and anxiety levels have changed?	No change	387 (45.5 %)	4/387 (1.0 %)	–	–	2/387 (0.5 %)	–	–
	Improved	69 (8.1 %)	3/69 (4.3 %)	**4.25 (0.79–20.5)**	**0.070**	3/69 (4.3 %)	**8.36 (1.32–66.0)**	**0.024**
	Worsened	394 (46.4 %)	72/394 (18.3 %)	**23.1 (9.20–78.3)**	**<0.001**	77/394 (19.5 %)	**47.6 (14.6–293)**	**<0.001**
Since the lockdown for COVID started, to what extent have you felt the things you are doing in your life are worthwhile?	Very worthwhile	281 (32.2 %)	5/270 (1.9 %)	–	–	8/270 (3.0 %)	–	–
	Somewhat worthwhile	446 (51.1 %)	26/432 (6.0 %)	**3.38 (1.37–10.2)**	**0.015**	33/432 (7.6 %)	**2.71 (1.27–6.50)**	**0.016**
	Somewhat unworthwhile	125 (14.3 %)	38/120 (31.7 %)	**27.6 (11.1–84.3)**	**<0.001**	34/120 (28.3 %)	**15.3 (6.90–38.1)**	**<0.001**
	Very unworthwhile	20 (2.3 %)	9/20 (45.0 %)	**48.7 (13.4–198)**	**<0.001**	6/20 (30.0 %)	**14.7 (4.03–52.7)**	**<0.001**
Since the lockdown for COVID started, how happy did you feel?	Very happy	96 (11.0 %)	1/93 (1.1 %)	–	–	1/93 (1.1 %)	–	–
	Somewhat happy	492 (56.4 %)	17/475 (3.6 %)	3.35 (0.65–61.7)	NS	20/475 (4.2 %)	4.03 (0.79–73.9)	NS
	Somewhat unhappy	250 (28.7 %)	42/242 (17.4 %)	**21.8 (4.41–396)**	**0.003**	39/242 (16.1 %)	**19.0 (3.84–346)**	**0.005**
	Very unhappy	34 (3.9 %)	18/32 (56.3 %)	**194 (32.3–3790)**	**<0.001**	20/32 (62.5 %)	**211 (35.4–4119)**	**<0.001**
Since the lockdown for COVID started, how satisfied have you been with your life?	Very satisfied	127 (14.5 %)	2/125 (1.6 %)	–	–	7/125 (5.6 %)	–	–
	Somewhat satisfied	381 (43.6 %)	12/366 (3.3 %)	1.78 (0.46–11.8)	NS	16/366 (4.4 %)	0.66 (0.27–1.81)	NS
	Somewhat dissatisfied	323 (37.0 %)	43/312 (13.8 %)	**10.3 (3.00–65.2)**	**0.002**	43/312 (13.8 %)	**2.55 (1.15–6.53)**	**0.019**
	Very dissatisfied	43 (4.8 %)	21/40 (52.5 %)	**88.8 (21.6–617)**	**<0.001**	15/40 (37.5 %)	**10.5 (3.75–32.2)**	**<0.001**
How much has prayer helped you cope with the way you have been feeling during the COVID lockdown?	Not at all	64 (7.3 %)	6/63 (9.5 %)	–	–	7/63 (11.1 %)	–	–
	Slightly	144 (16.5 %)	25/139 (18.0 %)	1.74 (0.68–5.10)	NS	23/139 (16.5 %)	1.42 (0.57–3.89)	NS
	Moderately	255 (29.2 %)	15/244 (6.1 %)	0.50 (0.19–1.51)	NS	17/244 (7.0 %)	0.57 (0.23–1.60)	NS
	A great deal	410 (47.0 %)	32/397 (8.1 %)	0.61 (0.24–1.77)	NS	34/397 8.6 %)	0.75 (0.31–2.01)	NS

NA – not applicable; NS – not significant (p > 0.10); OR – odds ratio; aOR – adjusted odds ratio; 95%CI – 95 % confidence interval.

Odds ratios are adjusted for sociodemographic variables: sex, age, highest educational level, religion, ethnicity, relationship status, place of residence and employment status.

a Includes loss of job/paid work of respondent or respondent's spouse, major cuts to household income, inability to pay bills/rent/mortgage, evicted/lost accommodation, unable to access sufficient food, unable to access medication, lost someone close (through COVID-19 or other illness), someone close currently in hospital (through COVID-19 or other illness).

### 3.4 Mental health and wellbeing

**Fig. 3:**
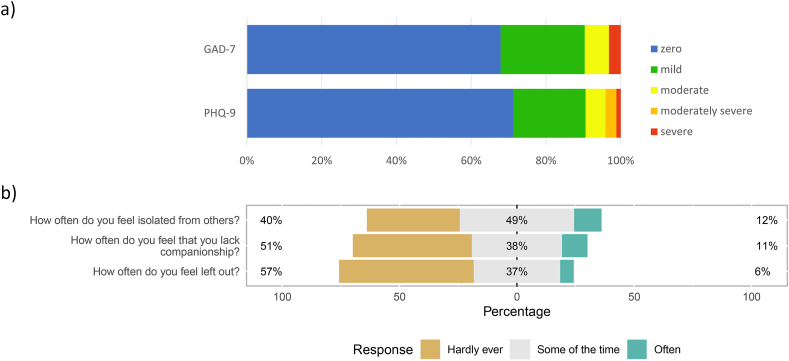
a) Distribution of anxiety (GAD-7) and depression (PHQ-9) scores of respondents and b) feelings of isolation reported by questionnaire respondents. GAD-7 – Generalised Anxiety Disorder 7 item instrument [16,17]; PHQ-9 – Patient Health Questionnaire 9 item instrument [15].

Of the 853 respondents completing all questions to derive PHQ-9 and GAD-7 scores, 165 (19.3 %) had mild, 45 (5.3 %) moderate, 25 (2.9 %) moderately severe and 10 (1.2 %) severe depression, and 192 (22.5 %) had mild, 55 (6.4 %) moderate and 27 (3.2 %) severe anxiety (Fig. 3a). Very few sociodemographic characteristics were significantly associated with moderate/severe depression or anxiety (Table 1). Older respondents were less likely to report moderate/severe anxiety (aOR for 35-64-year-olds and 65+ year-olds compared to 18-34-year-olds: 0.35 (95%CI 0.17–0.70) and 0.21 (95%CI 0.06–0.71), respectively). The same trend was seen for age and reporting moderate/severe depression, but this did not reach significance.

Single, never married respondents were about twice as likely to report depression and anxiety compared with those in a relationship and married/cohabiting (aOR 2.37 (95%CI 1.18–4.72) and 1.71 (95%CI 0.85–3.40), respectively). Part-time workers may have been less likely to report depression and anxiety than full-time workers (aOR 0.51 (95%CI 0.22–1.11) and 0.36 (95%CI 0.13–0.84), respectively), but this was only of borderline significance (p > 0.1 for depression, p = 0.026 for anxiety) and may be an artefact of multiple comparisons. Few other sociodemographic characteristics may have shown significant differences because of limitations in the diversity of the study population in terms of characteristics including religion, ethnicity and highest educational level.

An analysis of participants’ responses to each identified latent factor, expressed as standardised scores, against depression (PHQ-9) and anxiety (GAD-7) scores, is shown in Table S3 Supplementary Information File 1, with their respective correlation coefficients. The analysis suggests no association between any of the latent factors and depression or anxiety, except for a weakly positive association between missing activities: interests (missing going out for coffee, for meals, spending time in nature, exercising or going to the office) and identity (missing culture and community), and PHQ-9 and GAD-7 scores (Figs. S3b and S3g).

Table 3 reports changes in wellbeing reported by respondents since the start of the pandemic. Nearly half (46.4 %) reported that their mood and anxiety levels had worsened and 16.6 % reported that they felt the things they were doing in their lives were not worthwhile. 32.6 % reported feeling somewhat or very unhappy, while 41.8 % reported feeling somewhat or very dissatisfied with their life. PHQ-9 depression and GAD-7 anxiety scores followed the same pattern, with respondents that reported negatively regarding mood/anxiety, feeling worthwhile, happy and satisfied with life all having significantly higher odds of moderate/severe depression/anxiety scores, compared to those reporting more positively (aORs from 2.55 (95%CI 1.15–6.53) to 211 (95%CI 35.4–4119)). A large proportion of respondents reported feelings of isolation (61 % felt isolated from others, 49 % felt they lacked companionship and 43 % felt left out, some of the time or often, Fig. 3b), although changes in these feelings since lockdown were not recorded.

Changes in behaviour (smoking, alcohol consumption, volunteering) showed no association with depression/anxiety. However, both those reporting eating less (aOR depression 3.04 (95%CI 1.40–6.21), anxiety 1.88 (95%CI 0.86–3.80)) and more (aOR depression 4.34 (95%CI 2.61–7.31), anxiety 2.26 (95%CI 1.36–3.72)) since the start of the pandemic, were significantly more likely to report moderate/severe depression and anxiety than those reporting no change.

The vast majority of respondents (92.7 %) reported that prayer had helped them cope with the way they had felt during lockdown, with 29.2 % reporting that it helped moderately, and 47.0 % reporting that it helped a great deal (Table 3). While it did not reach statistical significance, those reporting that prayer helped moderately or a great deal were less likely to report moderate/severe depression or anxiety; *a priori* combining these two groups does suggest significantly lower depression and anxiety for those reporting that prayer helps with coping: aOR depression 0.37 (95%CI 0.22–0.63), anxiety 0.52 (95%CI 0.31–0.88), comparing those where prayer helps moderately/a great deal compared to those where prayer helps slightly/not at all.

## 4 Discussion

This survey aimed to improve our understanding of how changes in communal worship during the first UK lockdown may have affected the wellbeing and mental health of people active in faith residing in the UK through this time. Our study found levels of depression and anxiety among respondents higher than previously-reported population norms (prevalence of moderate/severe depression measured by PHQ-9: 9.4 % in our study versus 5.6 % in the general population pre-pandemic [21]; prevalence of moderate/severe anxiety measured by GAD-7: 9.6 % in our study versus 5 % pre-pandemic [22] – although these comparison data from nationally-representative surveys are from Germany, as to our knowledge no UK-based data are available). This increase in depression/anxiety is in agreement with a community cohort, UK-based study, which also found that older people were less likely to report psychological morbidity. However, while Jia et al. found increased risk of depression/anxiety for women, we found no such association [23].

Both factor analysis and thematic analysis of open-ended questions identified the distinction between religiosity and spirituality. In the latter, the importance of the social aspects of communal worship is recognised in addition to that of faith. Factor analysis also highlighted that missing multiple types of interests and participating in community activities had a negative impact on respondents’ mental health (interests and identity factors were positively associated with PHQ-9 and GAD-7 scores, albeit weakly). The experience of visiting physical places such as offices, gyms, hospitality and cultural venues and participating in community events could not be fully replaced by virtual contact.

This study also reinforced the importance of positive religious coping strategies, like prayer, in managing mental health during a crisis. A large majority (92.7 %) of respondents reported that prayer helped them cope with their feelings during the lockdown, with those reporting that prayer helped moderately or a great deal being significantly less likely to report moderate/severe depression or anxiety. The importance of personal prayer within the theory of religious coping has been identified previously [24]. By using a cognitive-behavioural framework (that links thoughts, emotions, and behaviours), the central importance of prayer may result from it containing mechanisms both for 1) providing a basis for guiding appraisals of life events (thinking through experience) and 2) that self-regulate thinking processes (i.e. that have a meditative function) [25]. The first of these allows individuals to make sense of their existence and contributes to an individual's self-perception, their own importance within the world, and the meaning and purpose behind life events [26]. The second, meditative prayer, may enable individuals to reduce self-focus, to engage mentally with stress, and therefore lower worry and rumination [26].

Religious coping can be divided into negative as well as positive religious coping. Positive religious coping primarily refers to having a sense of connectedness and a secure relationship with a loving God, while negative religious coping relates to feelings of being abandoned or punished by God and religious discontent [7,27]. Our findings regarding positive religious coping are in agreement with other studies. Village et al. reported that a positive change in spiritual wellbeing was positively correlated with both better mental and physical health during the third UK lockdown among members of the Church of England [28]. Lucchetti et al. reported that high use of religious and spiritual beliefs including private religious activities, religious attendance and spiritual growth during the pandemic in Brazil were associated with better mental health outcomes [29]. Pirutinsky et al. found that positive religious coping, intrinsic religiosity and trust in God were strongly correlated with less stress and more positive mental health impact in the American Orthodox Jewish population [30]. However, these authors also identified that negative religious coping and mistrust in God correlated with the inverse [30]. In addition, a survey of US Amazon Mechanical Turk workers suggested a stronger impact of negative religious coping than positive religious coping on COVID-19 related anxiety [31] and a study from Pakistan reported that primarily negative religious coping predicted depression and anxiety among caregivers [32]. Our UK-based survey did not ask questions relating to negative religious coping. However, 59 % of respondents provided additional information to open-ended questions (e.g., “If you would like to tell us anything else to help us with our study or give some feedback”), and we found no mention by respondents of comments relating to abandonment/punishment by God, or other related feelings, suggesting that negative religious coping was not prevalent among our study population.

### 4.1 Limitations

Despite considerable efforts to achieve diversity in terms of religion, ethnicity and other sociodemographic factors, our study sample was predominantly Christian (80.7 %), of white ethnicity (92.5 %) and highly educated (90.0 % had completed at least undergraduate-level education). Our recruitment strategy included directly linking with religious leaders of different faiths and engaging with social media, but this did not translate into uptake from a particularly diverse sample. It is therefore important to acknowledge that our findings may not be representative of all those active in faith in the UK, for every religion. The difficulty engaging with people of non-Christian religions may relate to the unprecedented situation during and after the first UK lockdown, where individuals may have been unavailable (for example, time constraints) or unwilling to complete an online survey on what many may regard as a very personal subject. Individuals within some faiths, such as the Haredi Jews, often have limited internet access, and therefore would have been unable to complete our online-only survey. Likewise, as only an English language survey version was offered, it would have excluded subjects who do not have a good proficiency in English. Given social distancing requirements in the UK at the time, we were unable to make face-to-face visits to religious leaders, places of communal worship and community groups, which may have increased visibility of the project to a diverse range of worshippers and increased trust and confidence for people to participate.

Respondents completed our cross-sectional survey shortly after the first UK lockdown. Self-reported behaviour change over the course of lockdown is therefore subject to recall and social desirability bias and the cross-sectional nature of the data does not allow for causal interpretation of our findings. As the survey was administered at a single time point, we were unable to measure how mental health and religious coping evolved throughout the pandemic and whether prevalence of moderate/severe anxiety and depression decreased once the worst effects of the pandemic in the UK were over, as observed by other studies [2,3]. A longitudinal study would allow a fuller understanding of the complexity in the ways that communal worship, the general dimensions of religiosity and spirituality, and religious coping impact mental health and wellbeing, including facilitating the use of religious coping scales [33,34] that assess the extent to which coping processed predict later wellbeing outcomes. However, our findings do still offer important insights into these relationships.

In our study we used open-ended questions to add context to the quantitative responses. This approach to incorporating some qualitative insights was used in other studies of religious practices during the pandemic, to support and illustrate the quantitative findings [35,36], and was cited by these authors as a strength of their studies, even though a formal qualitative analysis was not conducted.

### 4.2 Implications

These results could help shape any future update to the Covid-19 and pandemic preparedness policies around communal worship and singing for the UK [37] and elsewhere [38,39] by reinforcing the benefits of positive religious coping and integrating these effects into risk-benefit assessments of potential interventions. Our study did not focus on the harmful effects of negative religious coping, but nonetheless supports recommendations that religious leaders should focus on positive messaging and make all possible efforts to maintain contacts with worshippers (e.g., online services) to maintain the benefits of social contact and faith to improve mental health. Barriers to communal worship participation, including access to appropriate technology, need to be recognised and facilitators identified.

### 4.3 Conclusion

In summary, this study has provided valuable insights into the relationship between communal worship changes during the first UK lockdown and the mental health of faith-active individuals in the UK. The study found that positive religious coping mechanisms, such as prayer, played a significant role in helping individuals manage their mental health during this challenging period. While the study did not observe a gender disparity in the mental health impact of the lockdown, it did find that the loss of activities and interests had a detrimental effect on respondents' mental health. The results emphasise the necessity of considering both the spiritual and social aspects of communal worship in assessing the psychological impact of pandemic lockdowns and developing future public health interventions.

## CRediT authorship contribution statement

**Rebecca F. Baggaley:** Writing – review & editing, Writing – original draft, Formal analysis, Conceptualization. **Kai Man Alexander Ho:** Writing – review & editing, Writing – original draft, Conceptualization. **John Maltby:** Writing – review & editing, Writing – original draft, Formal analysis, Conceptualization. **Timothy C. Stone:** Writing – review & editing, Writing – original draft, Project administration, Investigation. **Áine Hoga:** Writing – review & editing, Writing – original draft, Project administration, Investigation. **Christopher Johnson:** Writing – review & editing, Writing – original draft, Visualization. **Robert Merrifield:** Writing – review & editing, Writing – original draft, Visualization. **Laurence B. Lovat:** Writing – review & editing, Writing – original draft, Resources, Investigation, Funding acquisition, Conceptualization.

## Declarations

This study was reviewed and approved by University College London Research Rthics Committee, with the approval number: 14223/002. All participants provided informed consent to participate in the study.

## Data availability statement

We have not deposited our dataset into a publicly available repository but data will be made available on request. The full dataset will not be shared but key variables, including the full set of open-ended responses, and not restricted to those used in the preparation of this manuscript, will be made available on reasonable request. Researchers who provide a methodologically sound proposal may access the full dataset after de-identification, directed to Professor Laurence Lovat: l.lovat@ucl.ac.uk. Data requestors will need to sign a data access agreement.

## Role of the funding source

The funders provided no role in study design; in the collection, analysis and interpretation of data; in the writing of the articles; and in the decision to submit it for publication. The views expressed are those of the authors and not necessarily those of the Wellcome Trust, the NIHR or the Department of Health and Social Care.

## Funding

This research was funded in part, by the 10.13039/100010269Wellcome Trust [203145Z/16/Z]. For the purpose of Open Access, the author has applied a CC BY public copyright licence to any Author Accepted Manuscript version arising from this submission. RFB was supported by an 10.13039/501100000272NIHR Advanced Fellowship (NIHR302494), a 10.13039/100010269Wellcome Trust Institutional Strategic Support Fund Fellowship (204801/Z/16/Z), the 10.13039/501100000272National Institute for Health and Care Research (NIHR) Applied Research Collaboration East Midlands (ARC EM) and Leicester 10.13039/501100000272NIHR Biomedical Research Centre (BRC). LBL is supported by the 10.13039/501100000272National Institute for Health Research 10.13039/501100012621University College London Hospitals Biomedical Research Centre, and by the Wellcome/EPSRC Centre for Interventional and Surgical Sciences (WEISS) [203145Z/16/Z] at UCL.

## Declaration of competing interest

The authors declare that they have no known competing financial interests or personal relationships that could have appeared to influence the work reported in this paper.
