# Supplementary material for: Impact of the COVID-19 pandemic on communal religious worshippers’ mental health and the benefits of positive religious coping
Source: Heliyon. 2024 Oct 9;10(21):e39093. doi: 10.1016/j.heliyon.2024.e39093 (PMC11550583; doi:10.1016/j.heliyon.2024.e39093)
Supplement: Multimedia component 1 [file mmc1.docx]

**Impact of the COVID-19 pandemic on communal religious worshippers’ mental health and the benefits of positive religious coping**

*Baggaley et al*

**Supplementary Information File 1**

**Factor Analysis**

We used factor analysis to collapse the data from the large number of questionnaire items on a) faith and b) missing various aspects of everyday life during lockdown, into a smaller number of variables (latent factors), for ease of interpretation. We explored the factor structures of a) the eight items relating to faith and b) the 12 items relating to missing activities using Exploratory Factor Analysis. We then used Confirmatory Factor Analysis (CFA) to verify any proposed factor structure emerging from the Exploratory Factor Analysis.

To perform factor analysis, we randomly split the sample data into two separate, equal datasets, one for Exploratory Factor Analysis and the second for Confirmatory Factor Analysis, ensuring the ratio of participants to variables exceeded the recommended 20:1, with minimum number of participants of 150 [1, 2]. We used the Kaiser-Meyer-Olkin test and Bartlett's test of sphericity to verify sampling adequacy. We used Exploratory Factor Analysis to allow any factor structure to emerge and properly assess the items’ latent factor without using any predefined model structure. To determine the number of factors from the Exploratory Factor Analysis, we used parallel analysis [3, 4]. Factors were then subjected to principal-axis factoring with promax rotation [5] (as it was expected that the factors would be correlated), with delta set to 0. Meaningful loadings were assessed using the criteria of .32 (*poor*), .45 (*fair*), .55 (*good*), .63 (*very good*), and .71 (*excellent*) , accepting every loading above .32 as relevant to the factor [6].

CFA was used to examine whether the emerging factor structure from the items identified in the Exploratory Factor Analysis could be replicated and presented as a good fit to the data. Satisfactory fit was assessed using goodness-of-fit indices including the Goodness of Fit Index (GFI), Normed Fit Index (NFI) and Adjusted Goodness of Fit Index (AGFI). Further details and justification for the factor analysis methods used can be found in the Supplementary Information Methods.

***Factor Analysis: missing activities***

Preliminary descriptive analyses of the items demonstrated that five of the 12 items had a positive skew (skewed variables from 1.16 to 2.81) and fell outside skewness statistics of +/-1 to suggest "very good" symmetry of a normal univariate distribution [7]. Therefore, we carried out a statistical analysis that did not assume the symmetry of a normal univariate distribution.

***Exploratory Factor Analysis***

The Kaiser-Meyer-Olkin measure of sampling adequacy of .74 and, in Bartlett's test of sphericity, x^2^=737.47, df=66, and p < .001, suggested that the sample size was satisfactory [8, 9]. From the original sample (n=939), 86 respondents had missing data on one or more of the 12 items relating to missing activities during the first COVID-19 lockdown, leaving 853 individuals included in the analysis. 427 individuals were included in the Exploratory Factor Analysis (EFA) and 426 in the Confirmatory Factor Analysis (CFA). The fourth eigenvalue (2.99, 1.24, 1.17 and 1.06) obtained from principal-axis factoring (due to the non-parametric nature of the data) failed to exceed the third eigenvalue (1.28, 1.21, 1.15 and 1.10) generated from a series of Monte Carlo simulations calculated for 12 variables and 427 participants from 1,000 randomly generated datasets, suggesting a two-factor solution.

CFA was used to examine whether the emerging factor structure from the nine items identified in the EFA could be replicated and presented as a good fit to the data. As the skewness and kurtosis for this sample also showed non-parametric properties (six of the items had a skew greater than +/- 1), we assessed the acceptability of the three-factor model to explore how well data fitted the model using unweighted least squares procedure is appropriate [10-14] The majority of these fit statistics (e.g., comparative ﬁt index, non-normed ﬁt index) are not provided with robust unweighted least squares procedures [15]. Therefore, satisfactory fit is indicated by other preferred goodness-of-fit indices, including the Goodness of Fit Index (GFI) and Normed Fit Index (NFI), having values above .95, and Adjusted Goodness of Fit Index (AGFI) having a value above .90 [16-20]. We found support for the factor-factor model with the GFI = .965 and AGFI = .983, with the NFI = .965, just falling short of the stated criteria. Therefore, we found some support for replication of the 3-factor structure of missing people close to the individual, missing social activities, and missing community activities.

***Faith***

Preliminary descriptive analyses of the items demonstrated that the data had a negative skew (*M* Skewness = -1.56; *M* Kurtosis = 3.28, highest skew = -3.01), and fell outside skewness statistics of +/-1 to suggest "very good" symmetry of a normal univariate distribution [7]. Therefore, we carried out a statistical analysis that did not assume the symmetry of a normal univariate distribution.

***Exploratory Factor Analysis (n=384)***

The Kaiser-Meyer-Olkin measure of sampling adequacy of .80 and, in Bartlett's test of sphericity, x^2^=1256.20, df=28, and p < .001, suggested that the sample size was satisfactory [8, 9]. 172 respondents had missing data on one or more of the eight items relating to faith, leaving 767 individuals included in the analysis. 384 individuals were included in the EFA and 383 in the CFA. The third eigenvalue (3.30, 1.89, and 0.77) obtained from maximum likelihood extraction failed to exceed the third eigenvalue (1.22, 1.14, and 1.07) generated from a series of Monte Carlo simulations calculated for eight variables and 384 participants from 1,000 randomly generated datasets, suggesting a two-factor solution. Factor Analysis results suggested two factors for faith item sets, which align with the well-established distinction between religiosity and spirituality (Table S2). The first factor contains four items relating to faith importance, engagement and purpose, aligned with coming together religiously and praying daily (orange: religiosity). The second factor contains three items relating to the importance of engaging in spiritual activities such as choir membership, singing and prayer (blue: spirituality). There was a small correlation between these two factors (r = .13) suggesting they are largely independent, accounting for no more than 2% of the shared variance.

***Confirmatory Factor Analysis* (n = 383).**

CFA was used to examine whether the emerging factor structure from the EFA could be replicated and presented as a good fit to the data. As the skewness and kurtosis for this sample also showed non-parametric properties (*M* Skewness = -1.36; *M* Kurtosis = 2.34, highest skew = -2.56), we assessed the acceptability of the two-factor model to explore how well data fitted the model using unweighted least squares procedure is appropriate (Hackett, 2019; Joreskog & Sorbom, 1989; Little, Cunningham, Shahar, & Widaman, 2002; Parry & McArdle, 1991; Wirth & Edwards, 2007). The majority of these fit statistics (e.g. comparative ﬁt index, non-normed ﬁt index) are not provided with robust unweighted least squares procedures (Blunch, 2013). Therefore, satisfactory fit is indicated by other preferred goodness-of-fit indices, including the Goodness of Fit Index (GFI) and Normed Fit Index (NFI), having values above .95, and Adjusted Goodness of Fit Index (AGFI) having a value above .90 (Byrne, 1998, 2010; Hu & Bentler, 1999; Kline, 2005; Schumacker & Lomax, 1996). We found some support for the two-factor model with the GFI = .974 and AGFI = .950, with the NFI = .942, just falling short of the stated criteria. Therefore, we found some support for replication of the 2-factor structure of religiosity and spirituality.

**Figure S1:** Region of UK residence of CONFESS questionnaire respondents.

**
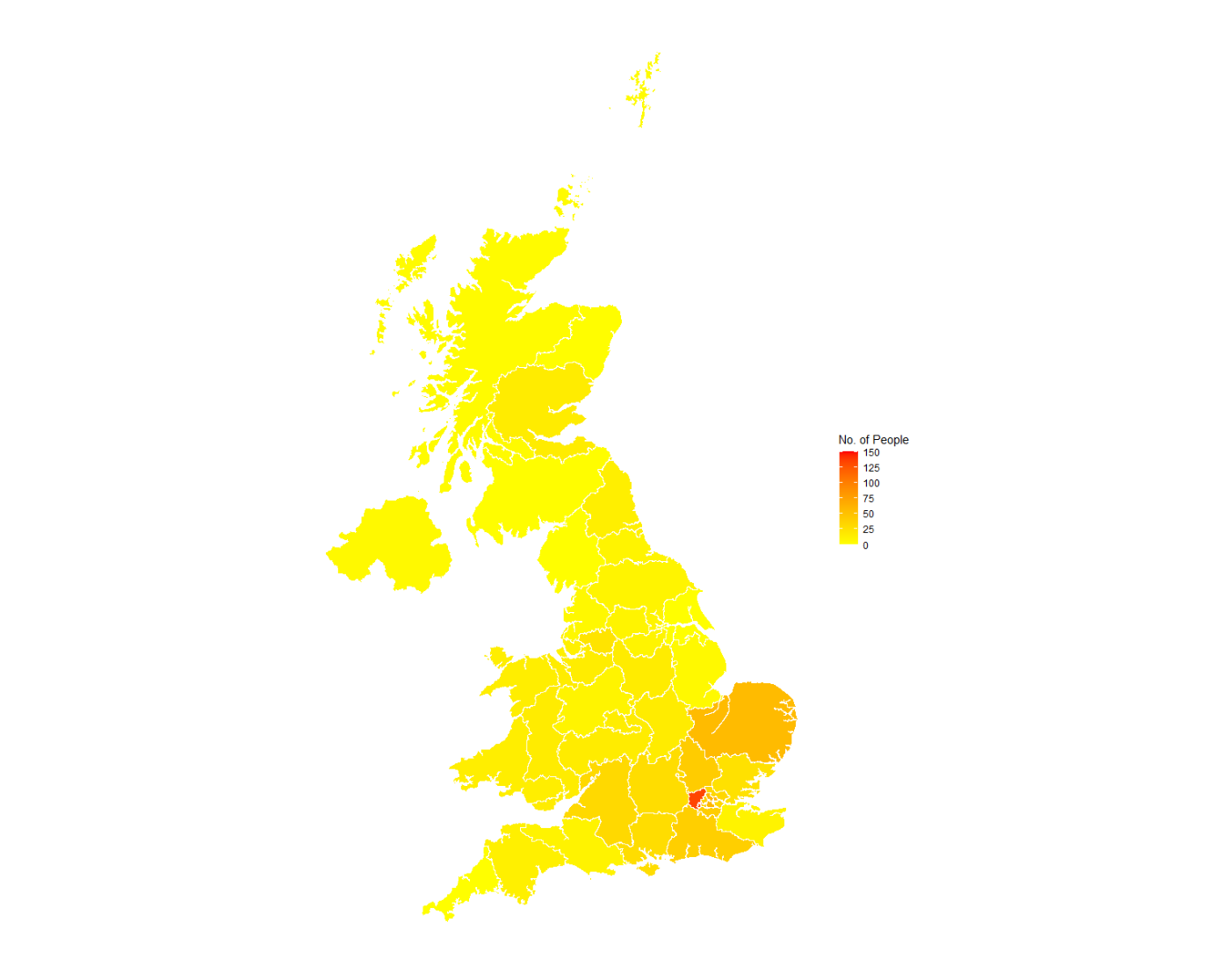
**

**Figure S2** Responses to questions on whether study participants were missing various aspects of life a lot or to some extent during the first UK COVID-19 lockdown, for a) females and b) males.

**Figure S3** Association between factors identified through factor analysis (missing activities: relationships, interests and identity and faith: religiosity and spirituality) and depression (PHQ-9, a-e) and anxiety (GAD-7 f-j) scores of respondents. For each factor, a score was calculated based on respondents’ answers to all questionnaire items associated with that factor. Scores for each factor were calculated as the additive sum of scores for all items. For missing activities factors, item scores were set as 0 for not missing an activity, 1 for missing it to some extent and 2 for missing it a lot. For faith factors, item scores were set as 0 for disagreeing or strongly disagreeing with each statement, 1 for agreeing and 2 for strongly agreeing. Factor scores were standardised to range from 0 to 10. Figures show boxplots for the distributions of depression/anxiety scores overlaid with the regression line for the two variables. Pearson’s Correlation Coefficient (r) shows the correlation between the two variables in each panel (0 to 1 positive correlation, 0 no correlation, 0 to -1 negative correlation). GAD-7 – Generalised Anxiety Disorder 7 item instrument [21, 22]; PHQ-9 – Patient Health Questionnaire 9 item instrument [23].

**Table S1:** Facebook groups on which the CONFESS Study was advertised.

| Acton Masjid | Babul Murad Centre | Bristol Hindu Temple | Buddhapadipa Temple |
| --- | --- | --- | --- |
| East London Mosque | Friendly Frummers | Hindu Temple Newcastle | Ilford Hindu Temple |
| Iskon Temple Watford | Islamic Integration Community | Jalaram Mandir Temple | Jamia Masjid |
| Jamyang Buddhist Centre | Jewish Connections | Junior Anglican Evangelical Conference | Kingsbury Buddhist Temple |
| London Asian Seventh-Day Adventist Church | London International Christian Church | London International Church @RhemaFaithMinistries | Mayfair Islamic Centre |
| Methodist Central Hall Westminster | MOO Modern Open Orthodox | Moslem Ali Khan Welfare Foundation | Slough Hindu Temple |
| Southampton Hindu Temple | Swindon Hindu Temple | Synagogues of London and the UK | UK Christian Events |
| UK Methodists | UKEvents.net | Vedic Society Hindu Temple |  |
| In addition, we advertised to various large mosques and synagogues around London and the UK. | | | |

**Table S2:** Patient Health Questionnaire 9 (PHQ-9) [23] and Generalised Anxiety Disorder Questionnaire 7 (GAD-7) [21, 24] items

| Over the last 2 weeks, how often have you been bothered by any of the following problems? | | |
| --- | --- | --- |
| **PHQ-9 item** |  |  |
| 1 | Little interest or pleasure in doing things? |  |
| 2 | Feeling down, depressed, or hopeless? |  |
| 3 | Trouble falling or staying asleep, or sleeping too much? |  |
| 4 | Feeling tired or having little energy? |  |
| 5 | Poor appetite or overeating? |  |
| 6 | Feeling bad about yourself - or that you are a failure or have let yourself or your family down? |  |
| 7 | Trouble concentrating on things, such as reading the newspaper or watching television? |  |
| 8 | Moving or speaking so slowly that other people could have noticed? Or the opposite - being so fidgety or restless that you have been moving around a lot more than usual |  |
| 9 | Thoughts that you would be better off dead, or of hurting yourself in some way? |  |
| **GAD-7 item** |  |  |
| 1 | Feeling nervous, anxious or on edge? |  |
| 2 | Not being able to stop or control worrying? |  |
| 3 | Worrying too much about different things? |  |
| 4 | Trouble relaxing? |  |
| 5 | Being so restless that it is hard to sit still? |  |
| 6 | Becoming easily annoyed or irritable? |  |
| 7 | Feeling afraid as if something awful might happen? |  |
| Responses: 0 = Not at all, 1 = Several days, 2 = More than half the days, 3 = Nearly every day | | |

**Table S3:** Factor analysis. A) aspects of life which respondents reported missing a lot or to some extent; and B) faith. For A), factors identified related to missing friends and family (orange: relationships), everyday activities (green: interests) and missing culture and community (blue: identity). For B), factors identified related to religiosity (orange) and spirituality (blue).

Results show principal-axis factoring with promax rotation. Factors were determined by parallel analysis. Loadings above .32 are indicated in bold.

| **Items** | **Factors** | | |
| --- | --- | --- | --- |
| 1. **Missing activities** |  | | |
|  | **1** | **2** | **3** |
| 1. Meeting up with family | **.61** | -.07 | -.05 |
| 1. Meeting up with friends | **.90** | -.19 | .02 |
| 1. Going out for coffee or drinks | .29 | **.46** | .01 |
| 1. Going out for meals | .25 | **.47** | .07 |
| 1. Going on holiday | .14 | .27 | .11 |
| 1. Going to cultural venues | -.04 | .26 | **.32** |
| 1. Taking part in community groups | -.04 | -.15 | **.86** |
| 1. Doing regular volunteering activities | .05 | .01 | .23 |
| 1. Spending time in nature | -.10 | **.54** | -.01 |
| 1. Going to the gym/other exercise | -.17 | **.48** | .02 |
| 1. Going to the office | -.01 | **.33** | -.01 |
| 1. Having time on your own | -.06 | .31 | -.10 |
| 1. **Faith** |  | | |
|  | **1** | **2** |  |
| 1. Physically meeting together regularly with members of my faith community is important to me | .371 | .254 |  |
| 1. I consider myself active in organised religion (usually going to church, temple, mosque etc) | **.744** | .019 |  |
| 1. Religious faith is extremely important to me | **.892** | -.026 |  |
| 1. I look to my religion to provide meaning and purpose in my life | **.867** | .017 |  |
| 1. I feel spiritually elevated when I am led in prayer by a band or choir | .039 | **.869** |  |
| 1. I pray or meditate daily | **.795** | -.089 |  |
| 1. I feel more spiritually elevated when I sing or chant prayers by myself than when I say them | .040 | **.483** |  |
| 1. I feel spiritually diminished when I am not led in prayer by a band or a choir | -.085 | **.652** |  |

**References**

1. Cattell RB. The Scientific Use of Factor Analysis in Behavioral and Life Sciences. New York: Plenum. <https://doi.org/10.1007/978-1-4684-2262-71978>.

2. Gorsuch RL. Factor Analysis, second edition, Hillsdale: Lawrence Erlbaum Associates. 1983.

3. Fabrigar LR, Wegener DT, MacCallum RC, Strahan EJ. Evaluating the use of exploratory factor analysis in psychological research. Psychological Methods. 1999;4(3):272–99. doi: <https://doi.org/10.1037/1082-989X.4.3.272>.

4. Ledesma RD, Valero-Mora P. Determining the Number of Factors to Retain in EFA: An easy-to-use computer program for carrying out Parallel Analysis. Available at: <https://scholarworks.umass.edu/pare/vol12/iss1/2> Accessed 7 April 2023. Practical Assessment, Research, and Evaluation. 2007;12 Article 2. doi: <https://doi.org/10.7275/wjnc-nm63>.

5. Osborne JW. What is Rotating in Exploratory Factor Analysis? Available from: <https://scholarworks.umass.edu/cgi/viewcontent.cgi?article=1251&context=pare> Accessed 6 July 2023. Practical Assessment, Research, and Evaluation. 2015;20(2).

6. Tabachnick BG, Fidell LS. Using multivariate statistics (6th ed.). Harlow: Pearson Education.2014.

7. Curran PJ, West SG, Finch JF. The robustness of test statistics to nonnormality and specification error in confirmatory factor analysis. Psychological Methods. 1996;1(1):16–29. doi: <https://doi.org/10.1037/1082-989X.1.1.16>.

8. Cerny CA, Kaiser HF. A study of a measure of sampling adequacy for factor-analytic correlation matrices. . Multivariate Behavioral Research. 1977;12(1):43-7.

9. Kaiser H. An index of factor simplicity. Psychometrika. 1974;39:31-6.

10. Hackett P. Quantitative research methods in consumer psychology : contemporary and data driven approaches. Routledge. 2019.

11. Joreskog KG, Sorbom D. LISREL 7: A Guide to the Program and Applications. SPSS. 1989.

12. Little TD, Cunningham WA, Shahar G, Widaman KF. To parcel or not to parcel: Exploring the question, weighing the merits. Structural Equation Modeling. 2002;9(2):151–73. doi: <https://doi.org/10.1207/S15328007SEM0902_1>.

13. Parry CDH, McArdle JJ. An Applied Comparison of Methods for Least- Squares Factor Analysis of Dichotomous Variables. Applied Psychological Measurement. 1991;15(1):35-46. doi: <https://doi.org/10.1177/014662169101500105>.

14. Wirth RJ, Edwards MC. Item factor analysis: Current approaches and future directions. . Psychological Methods. 2007;12(1):58-79. doi: <https://doi.org/10.1037/1082-989X.12.1.58>.

15. Blunch NJ. Introduction to structural equation modeling using SPSS and AMOS. Sage. 2013.

16. Byrne BM. Multivariate applications book series. Structural equation modeling with LISREL, PRELIS, and SIMPLIS: Basic concepts, applications, and programming. Lawrence Erlbaum Associates Publishers. 1998.

17. Byrne BM. Structural Equation Modeling with AMOS (2nd ed.). Routledge. 2010.

18. Hu LT, Bentler PM. Cutoff criteria for fit indices in covariance structure analysis: Conventional criteria versus new alternatives. Structural Equation Modeling, 6 SRC-G, 1–55. 1999.

19. Kline RB. Principles and practice of structural equation modeling (2nd ed.). Guilford Press. 2005.

20. Schumacker RE, Lomax RG. A beginner’s guide to Structural Equation Modelling. Lawrence Erlbaum Associates. 1996.

21. Spitzer RL, Kroenke K, Williams JB, Lowe B. A brief measure for assessing generalized anxiety disorder: the GAD-7. Arch Intern Med. 2006;166(10):1092-7. Epub 2006/05/24. doi: 10.1001/archinte.166.10.1092. PubMed PMID: 16717171.

22. Swinson RP. The GAD-7 scale was accurate for diagnosing generalised anxiety disorder. Evid Based Med. 2006;11(6):184. Epub 2007/01/11. doi: 10.1136/ebm.11.6.184. PubMed PMID: 17213178.

23. Kroenke K, Spitzer RL, Williams JB. The PHQ-9: validity of a brief depression severity measure. J Gen Intern Med. 2001;16(9):606-13. Epub 2001/09/15. doi: 10.1046/j.1525-1497.2001.016009606.x. PubMed PMID: 11556941; PubMed Central PMCID: PMCPMC1495268.

24. Lowe B, Decker O, Muller S, Brahler E, Schellberg D, Herzog W, et al. Validation and standardization of the Generalized Anxiety Disorder Screener (GAD-7) in the general population. Med Care. 2008;46(3):266-74. doi: 10.1097/MLR.0b013e318160d093. PubMed PMID: 18388841.
